# Sequential Synthesis Methodology Yielding Well-Defined Porous _75%_SrTiO_3_/_25%_NiFe_2_O_4_ Nanocomposite

**DOI:** 10.3390/nano12010138

**Published:** 2021-12-31

**Authors:** Ilyes Baba-Ahmed, Daniel Ghercă, Alexandra-Raluca Iordan, Mircea Nicolae Palamaru, Carmen Mita, Rachid Baghdad, Gabriel Ababei, Nicoleta Lupu, Mohamed Amine Benamar, Abdelkader Abderrahmane, Tiberiu Roman, Georgiana Bulai, Liviu Leontie, Adrian Iulian Borhan

**Affiliations:** 1 Laboratory of Fundamental and Applied Physics (FUNDAPL), Physics Department, Sciences Faculty, Saad Dahleb Blida 1 University, BP 270, Blida 09000, Algeria; baba.ing.ebm@gmail.com; 2 National Institute of Research and Development for Technical Physics, 47 Mangeron Boulevard, 700050 Iasi, Romania; dgherca@phys-iasi.ro (D.G.); gababei@phys-iasi.ro (G.A.); nicole@phys-iasi.ro (N.L.); 3 Faculty of Chemistry, Alexandru Ioan Cuza University of Iasi, 11 Carol I Boulevard, 700506 Iasi, Romania; alexandra.iordan@uaic.ro (A.-R.I.); palamaru@uaic.ro (M.N.P.); cmita@uaic.ro (C.M.); 4 Synthesis and Catalysis Laboratory, Matter Sciences Faculty, Ibn Khaldoun University of Tiaret, Tiaret 14000, Algeria; baghdadrachid@gmail.com; 5 Laboratory of Fundamental and Applied Physics (FUNDAPL), University Center of Tamenghasset, Amine Elokkal Elhadj Moussa Eg-Akhamouk, BP 10034, Sersouf, Tamanghasset 11000, Algeria; benamardz64dz@yahoo.fr; 6 Department of Electrical Engineering, Chosun University, 375, Seosuk-dong, Dong-gu, Gwangju 501759, Korea; abderrahmane.abdelkader@gmail.com; 7 Integrated Center of Environmental Science Studies in the North-Eastern Development Region (CERNESIM), Department of Exact and Natural Sciences, Institute of Interdisciplinary Research, Alexandru Ioan Cuza University of Iasi, 700506 Iasi, Romania; tiberiu.roman@chem.uaic.ro (T.R.); georgiana.bulai@uaic.ro (G.B.); 8 Faculty of Physics, Alexandru Ioan Cuza University of Iasi, 11 Carol I Boulevard, 700506 Iasi, Romania; lleontie@uaic.ro

**Keywords:** heterostructure, nanocomposite, porous foam SrTiO_3_/NiFe_2_O_4_, solid-state reaction, sol-gel auto-combustion

## Abstract

In this research, we reported on the formation of highly porous foam SrTiO_3_/NiFe_2_O_4_ (_100−x_STO/_x_NFO) heterostructure by joint solid-state and sol-gel auto-combustion techniques. The colloidal assembly process is discussed based on the weight ratio x (x = 0, 25, 50, 75, and 100 wt %) of NiFe_2_O_4_ in the _100−x_STO/_x_NFO system. We proposed a mechanism describing the highly porous framework formation involving the self-assembly of SrTiO_3_ due to the gelation process of the nickel ferrite. We used a series of spectrophotometric techniques, including powder X-ray diffraction (XRD), field emission scanning electron microscopy (FESEM), transmission electron microscopy (TEM), N_2_ adsorption isotherms method, UV-visible diffuse reflectance spectra (UV-Vis DRS), vibrating sample magnetometer (VSM), and dielectric measurements, to investigate the structural, morphological, optical, magnetic, and dielectric properties of the synthesized samples. As revealed by FE-SEM analysis and textural characteristics, SrTiO_3_-NiFe_2_O_4_ nanocomposite self-assembled into a porous foam with an internally well-defined porous structure. HRTEM characterization certifies the distinctive crystalline phases obtained and reveals that SrTiO_3_ and NiFe_2_O_4_ nanoparticles were closely connected. The specific magnetization, coercivity, and permittivity values are higher in the _75_STO/_25_NFO heterostructure and do not decrease proportionally to the amount of non-magnetic SrTiO_3_ present in the composition of samples.

## 1. Introduction

Nanomaterials such as nanoparticles, nanowires, nanotubes, nanorods, and quantum dots present unique optoelectronic, electrical, magnetic, and mechanical properties [1]. The development of new strategies to assemble these nanomaterials into patterned heterostructures with multiple functionalities and tailored physical properties is an urgent need for application in nanotechnological devices. The heterostructures offer attractive new possibilities for device applications due to the controlled integration of complementary nano-components, which exhibit synergistic effects that combine multiple functionalities in one structure [2,3]. The rapid advances in materials synthesis methods have made it possible to synthesize nanomaterials with well-defined sizes, shapes, structures, and morphologies. Although many heterostructure [4,5] systems have been reported, very few articles have shown the effect of morphology on the physical parameters [6]. Zheng et al. [1] explain that the assembling process of nanomaterials “is critically dependent on the nanostructure morphologies including domain patterns and shapes as well as structures and properties of the interfaces”. Starting from this concept, we present, in this study, how the colloidal assembly of NiFe_2_O_4_ nanoparticles can induce structural and morphological changes in a SrTiO_3_ matrix, that presents a superposition of cubic and tetragonal phases, depending on the weight ratio x of NiFe_2_O_4_. Moreover, the colloidal assembly of the attachment of the NiFe_2_O_4_ particles in the presence of seed SrTiO_3_ particles by using the sol-gel auto-combustion method leads to an open and porous foam morphology. The system SrTiO_3_/NiFe_2_O_4_ is known mostly for its capability as a visible-light-driven photocatalyst [7,8]. SrTiO_3_ (STO) is a prototypical cubic perovskite oxide with unique physical properties, such as wide bandgap, high dielectric constant, thermal stability, and large permittivity [9,10,11]. Besides, SrTiO_3_ is known as versatile powder support for the formation of heterostructures with specific properties for technological applications [12,13,14,15,16], because SrTiO_3_, as an intrinsic paraelectric, is able to stabilize ferroelectric order via substrate-induced strain, cation doping, ^18^O isotope substitutions, and defect engineering [17]. Hence, to induce ferromagnetic properties into SrTiO_3_, some researchers reported doping of STO using iron (Fe) [18], cobalt (Co) [19], manganese (Mn) [11] or by using non-magnetic sp-additives [12]. Recently, a ceramic composite of STO and nickel ferrite NiFe_2_O_4_ (NFO) has been reported to show ferroelectric, ferromagnetic, and magneto-dielectric properties [13]. NFO is a well-known nanomaterial with high Curie temperature, high chemical, and structural stability [14], having useful magnetic and electrical properties [8]. NFO is used in a wide range of applications including, electrical memory and switching devices [15], high-performance lithium-ion batteries [16], photocatalysis [20], and cancer therapy [21,22,23]. On the other hand, porous materials [24,25] have been obtained in the past decade [26,27] by various methods such as self-assembly of primary nanoparticles [28], combustion [29,30], smelting reaction [31], hydrothermal [32]. According to the literature [33], we tried here to extend the sol-gel auto-combustion method for the synthesis of porous heterostructure foams due to the possibility of controlling the chemical composition, homogeneity, morphology, shape, and phase composition of the materials. Unique and interesting features of SrTiO_3_/NiFe_2_O_4_ binary composite have gained currently great attention from many research studies [8,34]. Yongmei Xia et al. [34] showed from TEM images of SrTiO_3_/NiFe_2_O_4_ nanocomposites, that NiFe_2_O_4_ particles are uniformly dispersed onto the SrTiO_3_ without any accumulation, proving that the prepared nanocomposite is a uniform mixture of SrTiO_3_ and NiFe_2_O_4_ nanoparticles [34]. Moreover, in order to obtain hierarchical SrTiO_3_/NiFe_2_O_4_ composite nanostructures with an excellent light response, Panpan Jing et al. [8] used single-spinneret electrospinning and a side-by-side-spinneret electrospinning technique. In fact, the main advantage of these techniques is high productivity, but the formation of an uneven distribution of fiber diameter is difficult to avoid, while in the present study the uniformity of such nanocomposite is considered beneficial for our experimental ability to design useful photocatalytic properties in a rational way.

The results presented in this work aim to highlight a new topic and findings on the formation of the porous foam SrTiO_3_/NiFe_2_O_4_ (namely _100−x_STO/_x_NFO), heterostructure by joint solid-state reaction and sol-gel auto-combustion technique. The mechanism of colloidal assembly is discussed in the present study based on the weight ratio x (x = 0, 25, 50, 75, and 100 wt %) of NiFe_2_O_4_ in the _100−x_STO/_x_NFO system. The rational synthesis presented in this study, with emphasis on the colloidal assembly of the attachment of the NiFe_2_O_4_ particles in the presence of SrTiO_3_, represents a step forward in reaching multifunctional properties. These advances are at the core of progress in photocatalytic applications, including water remediation (dyes and pharmaceutical drugs degradation) and especially in water splitting (photocatalytic generation of H_2_). Thus, synthesized porous _75%_SrTiO_3_/_25%_NiFe_2_O_4_ nanocomposite materials offer flexibility for integrating multiple functionalities such as catalytic activity, adsorption capacity, photocatalytic activity, and magnetic properties that make them attractive for applications in photocatalysis.

## 2. Materials and Methods

### 2.1. Porous Heterostructure Foams _100−x_STO/_x_NFO Multistep Synthesis Method

Analytical grade precursors have been used to synthesize our materials, such as strontium carbonate (SrCO_3_, 99%, UCB chemicals; Brussels, Belgium), titanium dioxide (TiO_2_, 99%, Loba Feinchemie; Fischamend, Austria), nickel (II) nitrate (Ni (NO_3_)_2_·6H_2_O, 97%, Sigma Aldrich; Steinheim am Albuch, Baden-Württemberg, Germany), iron (III) nitrate (Fe (NO_3_)_3_·9H_2_O, 98%] Sigma Aldrich; Steinheim am Albuch, Baden-Württemberg, Germany), glycine (NH_2_CH_2_COOH, 99.7%, Merck; Steinheim am Albuch, Baden-Württemberg, Germany). First, the solid-state technique was used for the fabrication of the pristine SrTiO_3_ (STO) nanoparticles by mixing strontium carbonate and titanium dioxide with a molar ratio of 1:1. To ensure high homogeneity, the mixture was ground in an agate mortar for 6 h after adding a few drops of ethanol. Thereafter, the powder was calcined in air for 9 h at a temperature of 1000 °C. Pristine NiFe_2_O_4_ (NFO) were synthesized via a sol-gel auto-combustion method by mixing nickel nitrate, iron nitrate and glycine with a molar ration of 1:2:1 in 10 mL total volume of distilled water. The solution was agitated at speed of 300 rpm and temperature of 80 °C for 60 min until gelation occurs. Then, the as-prepared gel was heated on a sand bath for 6 h with a temperature-increasing step of 50 °C up to 350 °C, where the auto-ignition started suddenly, quickly, and violently with the flame. The typical experimental procedure used for the synthesis method is given as follows: first, the pristine synthetized STO powder is mixed with nickel nitrate, iron nitrate, and glycine with a molar ration of 1:2:1, in 10 mL of distilled water. Thereafter, gelation was achieved after 30 min under magnetic agitation of the solution at a speed of 300 rpm and temperature of 80 °C, followed by auto-ignition of the as-prepared gel in a sand bath for 6 h with a temperature increasing step of 50 °C up to 350 °C. In the end, the powder was kept for 9 h at a temperature of 1100 °C to obtain a stabilized structure. Thus, the newly heterostructures, designated as of _100−x_STO/_x_NFO, were obtained at a different mass percentage of NiFe_2_O_4_. Otherwise, to get insight over system design, primordially we consider the structural, magnetic, and optical properties translated in terms of morphology.

### 2.2. Porous Heterostructure _100−x_STO/_x_NFO Characterization

Phase identification and structural properties of starting nanomaterials calcined at 1000 °C and composite ceramics, obtained at 1100 °C, were determined by X-ray diffraction (Shimadzu LabX 6000; (Tokyo, Japan); diffractometer using Cu-Kα radiation (λ = 1.5406 Å)). The intensity and voltage of the X-ray source were set at 40 mA and 40 kV, respectively. The samples were scanned in reflection mode in the range 20–80° in 2θ with a step increment of 0.02° per step and a time per step of 0.2 s. Next, the morphology of the samples was studied using FE-SEM analysis, carried out on a Carl Zeiss NEON 40EsB; (Jena, Germany) with thermal Schottky field emission and accelerated Ga ions column. The FE-SEM micrographs were collected at different acceleration voltage and magnifications (1.8 kV, 50 kX 20 kV, 200 kX; 1.8 kV, 100 kX; and 5 kV, 150 kX). High-resolution electron micrographs of the calcined samples were taken by using a transmission electron microscope equipped with energy-dispersive X-ray spectroscopy (EDS) (Carl Zeiss LIBRA 200 MC UHR-TEM); (Jena, Germany) (at magnification of 700 kX and accelerating voltage HV of 200 kV. The samples for high resolution transmission electron microscopy (HR-TEM) were prepared by ultrasonically dispersing the powder in isopropanol and allowing a drop of this to dry on a carbon-coated copper grid. The specific surface area and the pore size of the calcined samples were measured by gas adsorption–desorption isotherms method, using nitrogen as adsorbate at 78 K on a Quantachrome Nova 2200 analyzer and the samples being degassed for 4 h at 250 °C before testing. UV-Vis absorption spectrum of the _75_STO/_25_NFO nanocomposite in the wavelength range of 200–1100 nm was investigated by using a Shimadzu UV-Vis spectrophotometer. The energy of the bandgap (Eg) was calculated using *Tauc’s* method [35]. The variation of magnetic properties of the composite ceramics was studied by hysteresis loop obtained using a vibrating sample magnetometer (VSM, Princeton/Lakeshore M3900, Lake Shore Cryotronics, Westerville, OH, USA)), at room temperature under a magnetic field in the range of ±10 kOe. Dielectric properties of the calcined samples, in a wide temperature range (20–200 °C) and for the frequency 2 ÷ 2 × 10^6^ Hz, were carried out on the Ag-electrodes applied to the polished faces of the pelletized samples by using an Agilent E4980A Precision LCR Meter (Santa Clara, CA, USA). The cylindrical pellets with a diameter of 12 mm and 1.5 mm thickness were obtained at 140 MPa by using a Hand Press (Carver Inc., Model 4350.L, Wabash, IN, USA) and thermal treatment of 3 h at 700 °C.

## 3. Results

### 3.1. Microstructural Characterization

**Figure 1:**
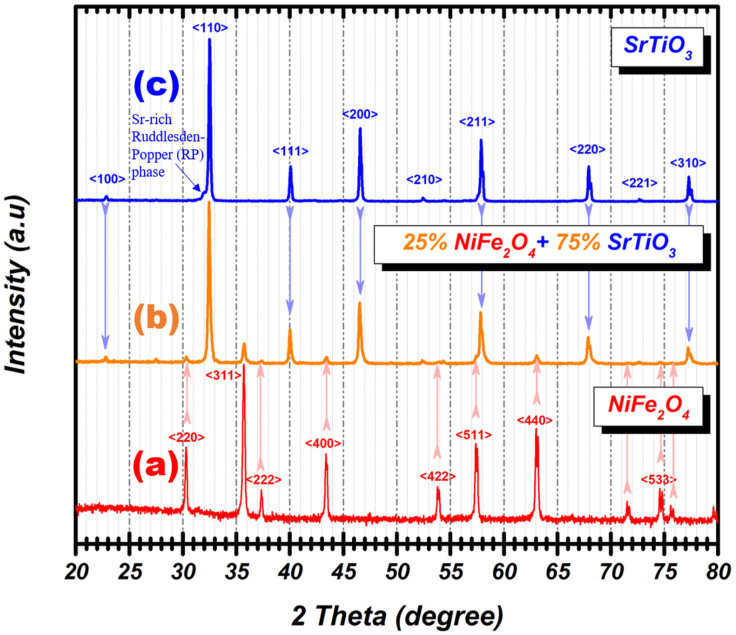
Powder XRD diffractograms of (**a**) pure cubic spinel phase NiFe_2_O_4_ synthesized by sol-gel auto-combustion method, (**b**) _75_STO/_25_NFO porous-foam nanocomposite formed by joint solid-state and sol-gel auto-combustion techniques and annealed at 1100 °C, and (**c**) pristine cubic perovskite phase SrTiO_3_ synthesized by solid-state technique by mixing strontium carbonate and titanium dioxide with a molar ratio of 1:1 and thermal treated for 9 h at 1000 °C.

The crystal structure and crystallinity of nanopowders, as pristine SrTiO_3_ and NiFe_2_O_4_, as well as _75_STO/_25_NFO porous-foam nanocomposite, were analyzed using X-ray diffraction analysis performed at room temperature in the 20–80° (2θ degree) range (Figure 1).

The diffraction peaks can be indexed to ICSD no. 98-009-1899 for SrTiO_3_ and to ICSD no. 98-016-5448 for NiFe_2_O_4_, obtained via a sol-gel auto-combustion method without any other impurity or secondary phases. The pristine SrTiO_3_ obtained by solid-state reaction presents a cubic structure and a Sr-rich Ruddlesden-Popper (RP) phase [36,37]. After combining simultaneously, the above two synthetic methodologies, _75_STO/_25_NFO porous-foam nanocomposite was obtained as presented in Figure 1b. The diffraction peak of both, perovskite and inverse spinel, respectively are tracked together in the powder XRD pattern of the obtained foam. Thus, the sequential synthesis methodology addressed in this study allows the formation of a novel porous foam nanocomposite _75_STO/_25_NFO. Compared with the cubic perovskite and the inverse spinel-type structures, the diffraction lines of both SrTiO_3_ and NiFe_2_O_4_ phases are present in the XRD pattern of the nanocomposites (Figure S1). The traditional crystallographic approach for structure determination (Rietveld refinement) was sufficient to confirm the crystal structure of the nanocomposites (Figures S2–S7). Rietveld analysis showed that the rutile TiO_2_ is present as a secondary phase because SrTiO_3_ is locally nonstoichiometric having Sr-rich RP phase. Using the normalized reference intensity ratio method (RIR), the percentage of rutile TiO_2_ phase in the _75_STO/_25_NFO porous-foam nanocomposite was determined to be about 2%.

According to data output from Rietveld refinement, as presented in Table 1, the crystallite size is 45 nm for NiFe_2_O_4_ and 49 nm for SrTiO_3_, respectively. In the case of composite, the Rietveld refinement was made by indexing the present phases, and their crystallite sizes were calculated by decomposing each corresponding diffraction peak, calculating FWHM. Therefore, the crystallite size values for NFO and STO were found to be 55 and 50 nm, respectively.

From the FE-SEM analysis of the _75_STO/_25_NFO porous-foam nanocomposite, the hierarchically porous material was formed by joint solid-state and sol-gel auto-combustion techniques. Hierarchical porosity is quite desirable for adsorption and photocatalytic processes and thus _75_STO/_25_NFO as visible-light-driven porous-foam nanocomposite with high contact surface area, high storage volume, ready mass transport, and a well-controlled porosity, which grant to this material a very high adsorption capacity and a high photocatalytic efficiency.

**Figure 2:**
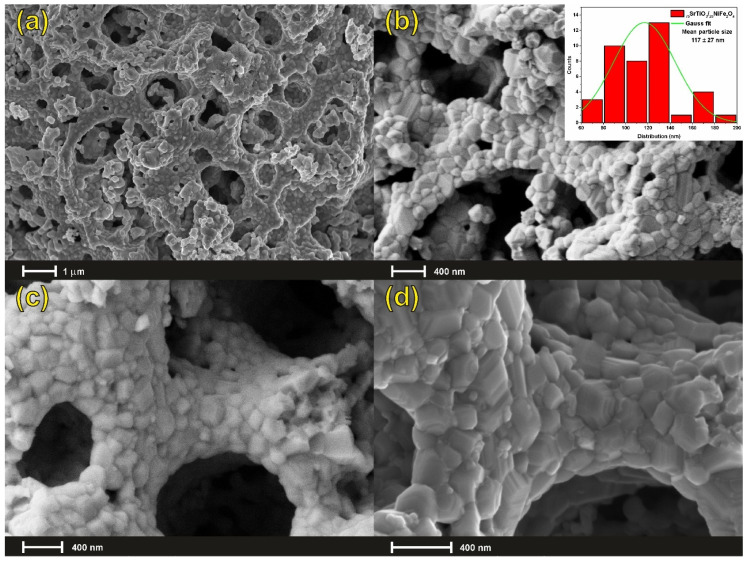
FE-SEM image of the porous structure (**a**) [scale 1 µm, electron high tension EHT = 5 KV and MAG = 10 kX], (**b**) [scale 400 nm, electron high tension EHT = 1.8 KV and MAG = 25 kX], (**c**) [scale 400 nm, electron high tension EHT = 5 kV and MAG = 30 kX], and (**d**) [scale 400 nm, electron high tension EHT = 5 KV and MAG = 45 kX], formed from the auto-combustion of hybrid 3D hierarchical xerogel composed of SrTiO_3_ nanoparticles, nickel, and iron glycinate. Histogram (b-inserted) showing the particle size distribution of the _75_STO/_25_NFO [SD: ±27 nm].

The pore formation mechanism in _75_STO/_25_NFO is shown in Figure 2. Particle interaction is dependent on sol-gel auto-combustion, which plays a key role in determining the morphology of the porous nanocomposite. Herein, a probable mechanism of pore formation in a highly porous framework is proposed and involves the self-assembly of SrTiO_3_ via the gelation process of the nickel nitrate, iron nitrate, and glycine. In the first step, polyhedral SrTiO_3_ nanoparticles were synthesized via the solid-state reaction of strontium carbonate and titanium dioxide with a molar ratio of 1:1 and thermally treated in the air for 9 h at a temperature of 1000 ºC. Next, these polyhedral SrTiO_3_ nanoparticles are used as a template to form a 3D hierarchical xerogel composed of nickel and iron glycinate at a temperature of 80 ºC for 60 min. Finally, the highly porous framework was converted to porous-foam _75_STO/_25_NFO nanocomposite on a sand bath for 6 h with a temperature-increasing step of 50 °C until auto-ignition occurs.

To understand the morphological evolution of the _75_STO/_25_NFO nanocomposite into a hierarchical porous-foam structure, composition-dependent experiments were performed.

**Figure 3:**
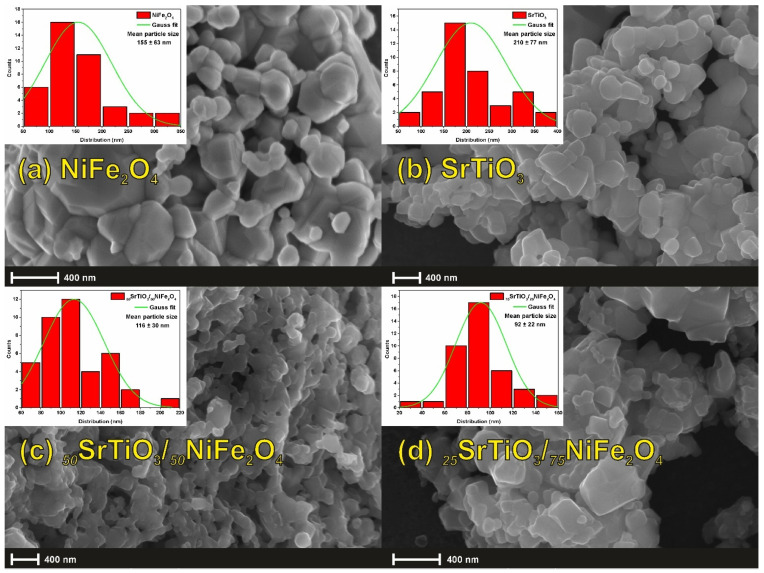
FE-SEM images of (**a**) pristine NiFe_2_O_4_ [scale 400 nm, electron high tension EHT = 50 KV and MAG = 35 kX], (**b**) pristine SrTiO_3_ [scale 400 nm, electron high tension EHT = 20 KV and MAG = 20 kX], (**c**) _50_STO/_50_NFO [scale 400 nm, electron high tension EHT = 20 KV and MAG = 25 kX], and (**d**) _25_STO/_75_NFO [scale 400 nm, electron high tension EHT = 20 KV and MAG = 35 kX] composites. Histograms (inserted) showing the particle size distribution of the starting materials and the two composites by using the ImageJ software. The mean value of the particle size and the errors (standard deviation (SD) were calculated by a Gaussian distribution applied to the histograms obtained.

Figure 3 depicts the FE-SEM images of the SrTiO_3_ template obtained from the solid-state reaction, pure NiFe_2_O_4_ via a sol-gel auto-combustion, and its composition-dependent morphological evolution following different weights ratio x = 0, 50, 75, and 100 wt % of NiFe_2_O_4_ in the _100−x_STO/_x_NFO system. Histograms, inserted in Figure 3, show the particle size distributions of the calcined starting materials and the _50_STO/_50_NFO and the _25_STO/_75_NFO. It is clear that with the NiFe_2_O_4_ increasing content in system, the average particle size decreases, due to the smaller particle size of the ferrite.

As seen in Figure 3b, the template consists of highly nonuniform polyhedral nanoparticles. When these polyhedral nanoparticles were reacted with different amounts of aqueous Ni^2+^ and Fe^3+^ solutions (concerning composition stoichiometry) at room temperature in the presence of glycine, they form nonhomogeneous frameworks. The framework becomes more and more disordered as the amount of NiFe_2_O_4_ increases, while highly porous-foam nanocomposite is obtained at the 3:1 mass ratio for SrTiO_3_:NiFe_2_O_4_ (Figure 2a–d), thereby indicating the optimal quantity of the reactants. Moreover, the selection of glycine, as fuel and chelating agent, in this synthesis was made according to its characteristic combustion temperature, because it affects the size of the crystallites, the structural stabilization, and the morphology. Glycine ignites at low temperatures, but the exothermic reaction is strong and violent with a large amount of gas released and high enthalpy leading to an increase in crystallites along with a good formation and high purity of the spinel-type structure. Our additional experiments reveal that when the concentration of the aqueous Ni^2+^ and Fe^3+^ glycinate was increased, the porous-foam-like structure partially collapsed to accommodate the formation of a nonhomogeneous system mixture of the two components.

**Figure 4:**
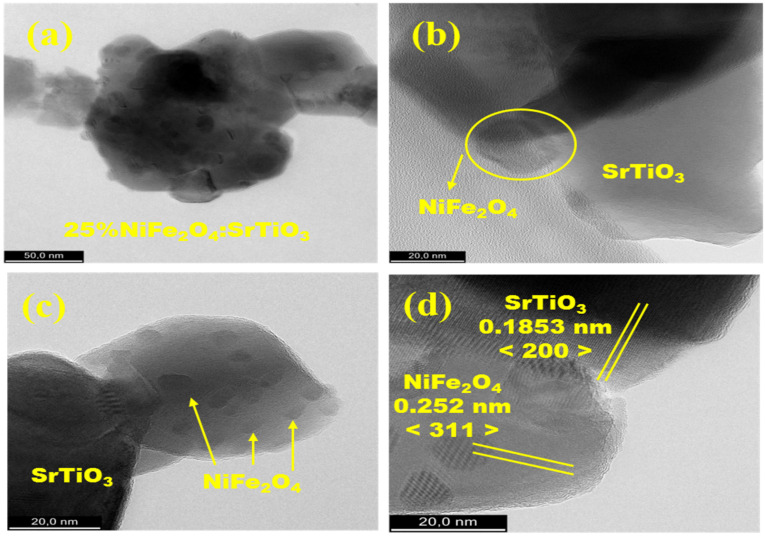
TEM image of the _75_STO/_25_NFO structure [scale 50nm] (**a**), HR-TEM image of the optimum holey interconnected NiFe_2_O_4_ nanoparticles with polyhedral SrTiO_3_ nanoparticles [scale 20 nm] (**b**–**d**).

To observe in more detail the morphology nature and lattice structure of _75_STO/_25_NFO, both transmission electron microscopy (TEM) and high-resolution TEM (HRTEM) imaging were performed. Figure 4a–d reveals the interconnected NiFe_2_O_4_ nanoparticles with polyhedral SrTiO_3_ nanoparticles leading to porous nature of _75_STO/_25_NFO nanocomposite, as well as another interesting feature of small NiFe_2_O_4_ particle attachments indicated by the yellow arrows present on the surface of the SrTiO_3_. Furthermore, it can be observed from Figure 4d that the optimum 3:1 ratio of components forms bulk chains rather than a core-shell type structure. The TEM energy dispersive spectroscopy (TEM-EDS) elemental mapping images of _75_STO/_25_NFO nanocomposite reveals the uniform distribution of Sr, Ti, and O atoms in the nanocomposite (Figure S8) while Fe and Ni atoms appear attached and among to the surface and interconnected respectively to the perovskite phase.

**Figure 5:**
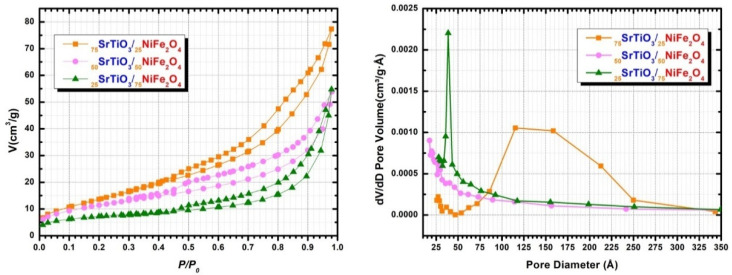
Nitrogen adsorption isotherms of the nanocomposites (**left**), and the corresponding average pore size distributions (**right**).

Figure 5 shows the nitrogen adsorption–desorption isotherms measurements of the composites and the corresponding average pore size distributions. It is found that all isotherms are of type IV with a H_3_ narrow hysteresis curve according to the IUPAC classification, characteristic of mesoporous materials (pores size in the range of 2.0–50.0 nm) [38]. The type of hysteresis loop provides information about the shape and connectivity of the inner pores [39], which plays an important role in the rate of gas adsorption. In our materials, H_3_ type hysteresis loops are associated with a porous structure that has pores with irregular size and shapes [40]. For all analyzed samples a gradual increase of the amount of N_2_ adsorbed from relatively small values of relative pressure is observed. However, the sudden increase in the amount of N_2_ adsorbed at low values of relative pressure, P/P_0_, proves that the samples present relatively large specific surface areas. Moreover, hysteresis loop at high relative pressures can be found, indicating the presence of porous structure, especially for the _75_STO/_25_NFO nanocomposite. The pore size distribution curve of _75_STO/_25_NFO shows a monomodal distribution with average pore diameters of between 13.97 and 15.86 nm (calculated from BJH desorption and adsorption isotherms, respectively) [41]. The _50_STO/_50_NFO exhibits a linear pore distribution with average pore diameter between 8.23 nm and 7.44 nm and the _25_STO/_75_NFO composite shows a narrow pore-size distribution with pore size of about 7.5 nm. Moreover, t-Plot micropore volume [42] is negative for all composites, meaning the samples do not contain micropores. The calculated textural characteristics of the samples, by using BET, BJH and t-plots methods, are summarized in Table 2. As expected, _75_STO/_25_NFO nanocomposite has the highest S_BET_ and Langmuir surface area, of 52 and 75 m^2^ g^−1^, respectively, as well as pore volume, suggesting that meso-pores make the largest contribution to the surface area. In this way, meso-porosity is produced in _75_STO/_25_NFO composite leading to an ordered porous structure, as confirmed by FE-SEM and HR-TEM. _50_STO/_50_NFO and _25_STO/_75_NFO composites showed smaller S_BET_ and pore volume, suggesting the entry of the nitrogen gas molecules was partially restricted [43]. Nearly double values of pore volume and diameter (0.148 nm and 15.86 nm, respectively) and larger size of specific surface area suggest that _75_STO/_25_NFO nanocomposite can provide more active sites and adsorb more reactive species in further photocatalytic experiments.

**Figure 6:**
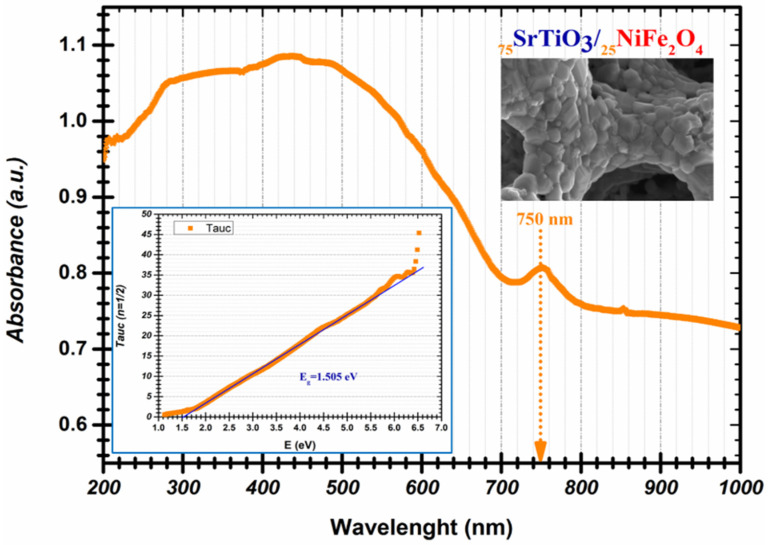
UV-vis DRS of _75_STO/_25_NFO nanocomposite as hierarchical porous-foam structure and (Inset) corresponding Tauc plot for bandgap determination.

Figure 6 shows the UV-Vis absorbance spectra of the _75_STO/_25_NFO nanocomposite as a hierarchical porous-foam structure to reveal the optical properties. It is very well studied in the literature that the absorption band of pure SrTiO_3_ sharply drops at about 388 nm whereas that of the pure NiFe_2_O_4_ continuously extends to the visible-light region (300 nm < λ < 1000 nm). This means that SrTiO_3_ only has a response to the UV light but the pure NiFe_2_O_4_ can respond to both the UV and visible light. Using Tauc’s relation: ((αhν)^2^ = A(hν-Eg)), the bandgap of 1.5 eV is determined by extrapolating the linear portion of the curve to the energy axis as shown in Figure 6 (inset). We can note that the _75_STO/_25_NFO nanocomposite with a hierarchical porous-foam structure has extended absorption spectra into the visible light region. Interestingly, it is noteworthy that the characteristic absorption peak of NiFe_2_O_4_ at about 750 nm still excites in that of synthesized hierarchical porous-foam and is certified further to the earlier reports about the similar composite nanoparticles and from this point of view, the heterojunctions of SrTiO_3_/NiFe_2_O_4_ bring up the complementarity that should exist between the two components (Table 3). Note that the study of these nanomaterials is a complex one, and here is presented only the first stage, in which we wanted to highlight an extremely interesting phenomenon of colloidal attachment of two reference nanomaterials in current technological and environmental applications.

**Figure 7:**
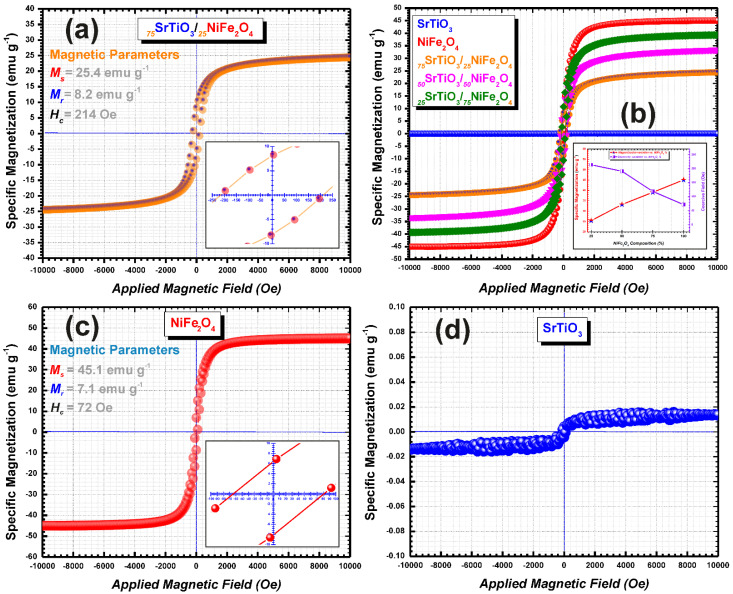
Room temperature hysteresis loops of the (**a**) _75_STO/_25_NFO nanostructure, (**b**) all composites synthesized with different spinel phase percentage together with inset graph showing the magnetization and coercivity variation with spinel phase ratios, (**c**) pristine NiFe_2_O_4_ nanoparticles with inset graph showing the loop at near-zero magnetic field, and (**d**) pristine SrTiO_3_ nanoparticles.

Room temperature hysteresis loops of our pure and composite materials are represented in Figure 7. Indeed, as shown in Figure 7d the recorded room temperature hysteresis loops reveal that the pure SrTiO_3_ has no magnetism; however, pristine NiFe_2_O_4_ nanoparticles show a ferromagnetic behavior with the saturation magnetization (M_S_) of about 45 emu g^−1^ (Figure 7c).

Considering the presence of the non-magnetic SrTiO_3_, the $M_{S}$ values for the synthesized composites with different ratios of NiFe_2_O_4_ from 25% up to 75% (Figure 7b), are lower than that of the pure spinel phase and they are between 25 and 39 emu g^−1^ (see Table 4). It is interesting to note that the magnetization does not decrease proportionally to the amount of non-magnetic SrTiO_3_ present in the composition of samples (Figure 7b). Moreover, the highest coercivity value is determined for the _75_STO/_25_NFO sample, maybe due to the high magneto crystalline anisotropy, emphasizing the same trend described vide-supra. The mentioned phenomenon needs more experimental work corroborated with theoretical simulation to unequivocally assess the magnetic properties of the as-prepared nanocomposites in hierarchical assembly as well in nonhomogeneous form.

### 3.2. Comparative Analysis of the Dielectric Properties

**Figure 8:**
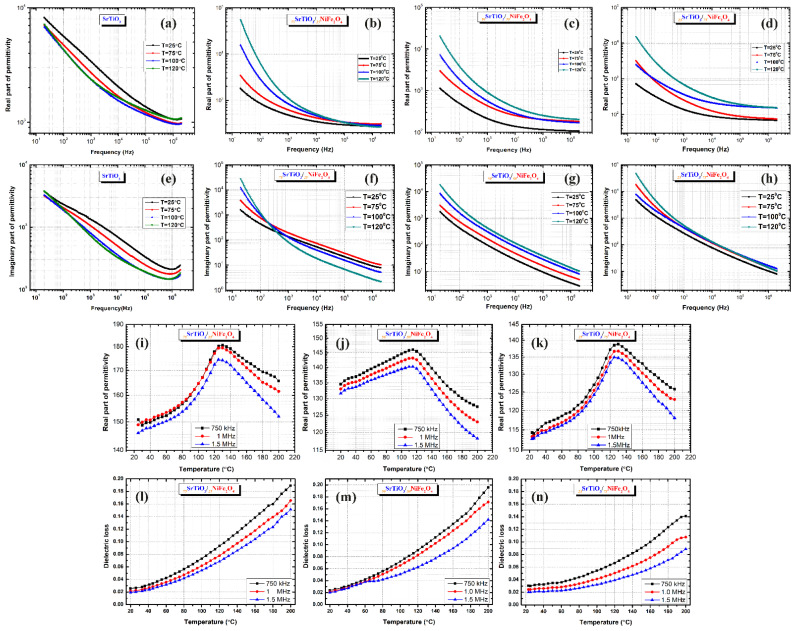
Dielectric dispersion at few temperatures of the materials: (**a**,**e**) real and imaginary parts of permittivity of the SrTiO_3_; and (**b**–**d**) real part of permittivity and (**f**–**h**) imaginary part of permittivity of _100−x_STO/_x_NFO composites. Temperature dependence of the dielectric characteristics at a few selected frequencies for _100−x_STO/_x_NFO composites: (**i**–**k**) permittivity; (**l**–**n**) tangent loss.

The evolution with the frequency of the real and imaginary parts of permittivity for the starting material and the composites prepared is comparatively presented in Figure 8a–h. For a better understanding of the extrinsic permittivity of the composite materials, SrTiO_3_ was prepared under the same conditions as the composites for the dielectric analysis. The frequency dependence of real and imaginary part of permittivity of pure pristine SrTiO_3_ ceramic at few temperatures are shown in Figure 8a,e, showing a different behavior compared to that of composites. At low frequency, the sample presents a strong decay and at high frequency (10^5^–10^6^) Hz, where all the extrinsic phenomena were canceled, the permittivity tends to its intrinsic values ~1100.

On the other hand, the real part of permittivity vs. frequency indicates rather similar dielectric behaviors for all composites. The frequency dispersion is higher with increasing temperature and this increase is enhanced at lower frequencies. The _75_STO/_25_NFO composite shows higher permittivity at low frequencies, increasing with temperature. For example, at f = 1 kHz, the permittivity assumes values of about 457 at 25 °C and 1072 at 120 °C for the _75_STO/_25_NFO, while for the _25_STO/_75_NFO composite, of about 131 and 710 at the same temperatures, respectively. Imaginary parts of permittivity (Figure 8f–h) show a strong decrease with frequency from 2 × 10^4^ to ~2 for _75_STO/_25_NFO and 3 × 10^4^ to 10 for _25_STO/_75_NFO composites, respectively, at the frequency between 20 Hz to 10^6^ Hz and 120 °C. Both composites show a low-frequency decay of the real and imaginary part of permittivity, which strongly increases with temperature. However, apart from the linear variation observed in the case of _50_STO/_50_NFO composite, the two materials appear to be temperature independent with increasing frequency and temperature. While for _50_STO/_50_NFO composite, these extrinsic mechanisms seem to be canceled for frequency higher than 10^5^ Hz, in case of the _75_STO/_25_NFO and _25_STO/_75_NFO composites, they continue to exist in all investigated frequency range. The observed differences show that extrinsic contributions are slightly different in these types of composites. The extrinsic contributions are due to Maxwell–Wagner relaxations and dc-conductivity caused by the slow-charged species activated at low frequencies and high temperatures [46]. The Maxwell–Wagner relaxation is related to inhomogeneities and interfaces [47], and it seems that the better dielectric characteristics obtained for slightly temperature-dependent _75_STO/_25_NFO composite are a consequence of different microstructural properties, e.g., hierarchical porous-foam morphology by particle attachment. At high frequency (around 10^5^ Hz), where the charge defect-associated relaxations are no longer active, the permittivity tends to its intrinsic value [48,49]. For this frequency, at 120 °C, values around ~ 320 for _75_STO/_25_NFO (independent with temperature), ~250 for _75_STO/_25_NFO (temperature-dependent) and ~190 for _25_STO/_75_NFO (temperature dependent), are found. The dielectric losses at high frequencies (1 MHz) (Figure 8l–n) seem to be independent of the composite type, temperature-dependent and are in the range of 0.02–0.18 for temperatures between 20 and 200 °C.

The temperature dependence of permittivity shows some differences for the composites according to the composition (Figure 8i–k). A broad permittivity maximum can be detected in the range of about 120–140 °C for all composites and it originates from a relaxor character or a reduction of ferroelectric character [50,51]. The shape and the position of the permittivity maximum are affected by an extrinsic dielectric effect that tends to cover the intrinsic ferroelectric behavior in the composites and the permittivity decreases with temperature and frequency. The shape of the permittivity maximum might be explained by local compositional variations and morphological differences [52].

## 4. Conclusions

Sequential synthesis methodology addressed in this study enables the successful fabrication of _75_STO/_25_NFO as porous-foam in nature by joint solid-state and sol-gel auto-combustion technique. The diffraction peak of both, perovskite and inverse spinel, respectively, were tracked together in the powder XRD pattern of the obtained composite. The _75_STO/_25_NFO composite exhibits a powerful visible light response with a band gap energy of 1.505 eV and a value of the room temperature permittivity of 457. In addition, _75_STO/_25_NFO composite shows higher permittivity at low frequencies, which is proportional to the temperature increase. Morphological analyses performed by FE-SEM and HRTEM reveal that the consolidation of particles occurs by exact stacking of NiFe_2_O_4_ particles along crystal facets of SrTiO_3_. _75_STO/_25_NFO nanocomposite showed the highest S_BET_ and Langmuir surface area values, of 52 and 75 m^2^ g^−1^, respectively, as well as pore volume, which can suggest that meso-pores make the largest contribution to the surface area. The values of the specific magnetization increased from 25.4 to 45.1 emu g^−1^ when the ratio x in _100−x_STO/_x_NFO increases from 25 to 100. The coercivity showed the highest value of 214 Oe for the _75_STO/_25_NFO sample. The nanocomposite can be considered as the famous heterojunction connection by sequential synthesis methodology addressed in this study with emphasis on the colloidal assembly of the attachment of the NiFe_2_O_4_ particles in the presence of SrTiO_3_.

## Figures and Tables

**Table 1:** Determined Cell Parameters after Rietveld refinement.

Sample	Space Group	Direct Cell Parameters (Å)	Direct Cell Volume (Å^3^)	Reliability Factors
SrTiO_3_	*P m -3 m*	*a* = *b* = *c* = 3.9034(3)	59.476(3)	0.796/0.899
NiFe_2_O_4_	*F d -3 m*	*a* = *b* = *c* = 8.3402(3)	580.141(4)	1.79/3.97
TiO_2_	*P 42/m n m*	*a* = *b* = 4.5912(2) *c* = 2.9697(6)	62.601(4)	5.01/13.2

**Table 2:** Textural characteristic of the obtained nanocomposites.

Sample	BET Surface Area (m^2^ g^−1^)	Langmuir Surface Area (m^2^ g^−1^)	Volume of Pores (Adsorption) (cm^3^ g^−1^)	Volume of Pores (Desorption) (cm^3^ g^−1^)	Average Pore Width (Adsorption) (nm)	Average Pore Width (Desorption) (nm)
_75_STO/_25_NFO	52 ± 1	75 ± 1	0.148 ± 0.004	0.146 ± 0.004	15.86 ± 0.91	13.97 ± 0.78
_50_STO/_50_NFO	39 ± 1	59 ± 3	0.082 ± 0.001	0.074± 0.003	8.30 ± 0.77	7.44 ± 0.97
_25_STO/_75_NFO	25 ± 2	36 ± 1	0.078 ± 0.002	0.074 ± 0.006	7.76 ± 1.12	7.36 ± 0.64

**Table 3:** Bandgap values of NiFe_2_O_4_, SrTiO_3_ and SrTiO_3_/NiFe_2_O_4_ composite thereof.

Compound	Composition	Band Gap Values (eV)	References
SrTiO_3_	pristine	3.20	[7]
SrTiO_3_	pristine	3.15	[34]
NiFe_2_O_4_	pristine	1.70	[44]
NiFe_2_O_4_	pristine	1.82	[34]
NiFe_2_O_4_	pristine	1.40	[45]
γ-Fe_2_O_3_@NiFe_2_O_4_	50% γ-Fe_2_O_3_ 50% NiFe_2_O_4_	1.38	[45]
SrTiO_3_/NiFe_2_O_4_	15% SrTiO_3_ 85% NiFe_2_O_4_	1.42	[34]
_75_STO/_25_NFO	75% SrTiO_3_ 25% NiFe_2_O_4_	1.50	This Study

**Table 4:** Magnetic properties of NiFe_2_O_4_, SrTiO_3_, and SrTiO_3_/NiFe_2_O_4_ composites thereof.

Sample	Specific Magnetization (emu g^−1^)	Remanent Magnetization (emu g^−1^)	Coercive Field (Oe)	References
SrTiO_3_	-	-	-	This Study
_75_SrTiO_3_/_25_NiFe_2_O_4_	25.4	8.2	214	This Study
_50_SrTiO_3_/_50_NiFe_2_O_4_	33.2	10.5	191	This Study
_25_SrTiO_3_/_75_NiFe_2_O_4_	39.1	10.2	119	This Study
NiFe_2_O_4_	45.1	7.1	72	This Study
SrTiO_3_/NiFe_2_O_4_ porous nanotubes	10	n.a	n.a	[8]
SrTiO_3_/NiFe_2_O_4_ nanoparticle-in nanotubes	18	n.a	n.a	[8]
NiFe_2_O_4_	40	n.a	n.a	[34]
85% NiFe_2_O_4_ 15% SrTiO_3_	23.3	n.a	n.a	[34]

## Data Availability

The data presented in this study are available from the corresponding author on request.

## Supplementary Materials

The following supporting information can be downloaded at: https://www.mdpi.com/article/10.3390/nano12010138/s1, Figure S1. The superimposed XRD patterns of perovskite phases (SrTiO_3_) synthesized with different ratios of spinel phase (NiFe_2_O_4_); Figure S2. The Rietveld refinement of the _75_STO/_25_NFO. Black solid line is the best fits to the experimental data, the blue line represents the differences between the experimental and calculated data while the vertical markers represent the Bragg peaks positions in blue for Cubic perovskite phase, in red for Cubic spinel phase and in green for Tetragonal TiO_2_ phase; Figure S3. Le Bail refinement from laboratory XRD patterns at RT The Rietveld refinement of SrTiO_3_ nanoparticles. Black solid line is the best fits to the experimental data, the blue line represents the differences between the experimental and calculated data while the vertical markers represent the Bragg peaks positions in blue for Cubic perovskite phase and in red for Ruddlesden–Popper phase; Figure S4. The Rietveld refinement of SrTiO_3_ with 25% of NiFe_2_O_4_. Black solid line is the best fits to the experimental data, the blue line represents the differences between the experimental and calculated data while the vertical markers represent the Bragg peaks positions in blue for Cubic perovskite phase and in red for Cubic spinel phase and in green for Tetragonal TiO_2_ phase; Figure S5. The Rietveld refinement of SrTiO_3_ with 50% of NiFe_2_O_4_. Black solid line is the best fits to the experimental data, the blue line represents the differences between the experimental and calculated data while the vertical markers represent the Bragg peaks positions in blue for Cubic perovskite phase, in green for Tetragonal perovskite phase and in red Cubic spinel phase; Figure S6. The Rietveld refinement of SrTiO_3_ with 75% of NiFe_2_O_4_. Black solid line is the best fits to the experimental data, the blue line represents the differences between the experimental and calculated data while the vertical markers represent the Bragg peaks positions in blue for Cubic perovskite phase, in green for Tetragonal perovskite phase and in red Cubic spinel phase; Figure S7. The Rietveld refinement of NiFe_2_O_4_ nanoparticles. Black solid line is the best fits to the experimental data, the blue line represents the differences between the experimental and calculated data while the vertical markers represent the Bragg peaks positions in blue for Cubic spinel phase; Figure S8. TEM dark field image of NiFe_2_O_4_ particle attachment on SrTiO_3_ polyhedral nanoparticles (a) and the corresponding elemental mapping for all elements Fe, Ni, Sr, Ti, O (b) Sr, Ti (c) and Fe, Ni (d)**.**

## References

[1] Zheng H., Zhan Q., Zavaliche F., Sherburne M., Straub F., Cruz M.P., Chen L.-Q., Dahmen U., Ramesh R. (2006). Controlling self-assembled perovskite− spinel nanostructures. Nano Lett..

[2] Lendlein A., Trask R.S. (2018). Multifunctional materials: Concepts, function-structure relationships, knowledge-based design, translational materials research. Multifunct. Mater..

[3] Gibson R.F. (2010). A review of recent research on mechanics of multifunctional composite materials and structures. Compos. Struct..

[4] Ren K., Zheng R., Xu P., Cheng D., Huo W., Yu J., Zhang Z., Sun Q. (2021). Electronic and Optical Properties of Atomic-Scale Heterostructure Based on MXene and MN (M= Al, Ga): A DFT Investigation. Nanomaterials.

[5] Ren K., Sun M., Luo Y., Wang S., Yu J., Tang W. (2019). First-principle study of electronic and optical properties of two-dimensional materials-based heterostructures based on transition metal dichalcogenides and boron phosphide. Appl. Surf. Sci..

[6] Chen Y., Lai Z., Zhang X., Fan Z., He Q., Tan C., Zhang H. (2020). Phase engineering of nanomaterials. Nat. Rev. Chem..

[7] Reunchan P., Ouyang S., Umezawa N., Xu H., Zhang Y., Ye J. (2013). Theoretical design of highly active SrTiO 3-based photocatalysts by a codoping scheme towards solar energy utilization for hydrogen production. J. Mater. Chem. A.

[8] Jing P., Du J., Wang J., Lan W., Pan L., Li J., Wei J., Cao D., Zhang X., Zhao C. (2015). Hierarchical SrTiO_3_/NiFe_2_O_4_ composite nanostructures with excellent light response and magnetic performance synthesized toward enhanced photocatalytic activity. Nanoscale.

[9] Vivek S., Geetha P., Saravanan V., Kumar A.S., Lekha C.S.C., Sudheendran K., Anantharaman M.R., Nair S.S. (2019). Magnetoelectric coupling in strained strontium titanate and Metglas based magnetoelectric trilayer. J. Alloys Compd..

[10] Coey J.M.D., Venkatesan M., Stamenov P. (2016). Surface magnetism of strontium titanate. J. Phys. Condens. Matter.

[11] Kleemann W., Dec J., Tkach A., Vilarinho P.M. (2020). SrTiO_3_—Glimpses of an Inexhaustible Source of Novel Solid State Phenomena. Condens. Matter.

[12] Bannikov V.V., Shein I.R., Kozhevnikov V.L., Ivanovskii A.L. (2008). Magnetism without magnetic ions in non-magnetic perovskites SrTiO_3_, SrZrO_3_ and SrSnO_3_. J. Magn. Magn. Mater..

[13] Ke H., Zhang H., Zhou J., Jia D., Zhou Y. (2019). Room-temperature multiferroic and magnetodielectric properties of SrTiO_3_/NiFe_2_O_4_ composite ceramics. Ceram. Int..

[14] Jian G., Xue F., Zhang C., Yan C., Zhao N., Wong C.P. (2017). Orientation dependence of elastic and piezomagnetic properties in NiFe_2_O_4_. J. Magn. Magn. Mater..

[15] Dey P., Debnath R., Singh S., Mandal S.K., Roy J.N. (2017). Irreversibility in room temperature current–voltage characteristics of NiFe_2_O_4_ nanoparticles: A signature of electrical memory effect. J. Magn. Magn. Mater..

[16] Jin R., Jiang H., Sun Y., Ma Y., Li H., Chen G. (2016). Fabrication of NiFe_2_O_4_/C hollow spheres constructed by mesoporous nanospheres for high-performance lithium-ion batteries. Chem. Eng. J..

[17] Xu R., Huang J., Barnard E.S., Hong S.S., Singh P., Wong E.K., Jansen T., Harbola V., Xiao J., Wang B.Y. (2020). Strain-induced room-temperature ferroelectricity in SrTiO_3_ membranes. Nat. Commun..

[18] He J., Lu X., Zhu W., Hou Y., Ti R., Huang F., Lu X., Xu T., Su J., Zhu J. (2015). Induction and control of room-temperature ferromagnetism in dilute Fe-doped SrTiO_3_ ceramics. Appl. Phys. Lett..

[19] Zhang W., Li H.-P., Pan W. (2012). Ferromagnetism in electrospun Co-doped SrTiO_3_ nanofibers. J. Mater. Sci..

[20] Zhu H.-Y., Jiang R., Fu Y.-Q., Li R.-R., Yao J., Jiang S.-T. (2016). Novel multifunctional NiFe_2_O_4_/ZnO hybrids for dye removal by adsorption, photocatalysis and magnetic separation. Appl. Surf. Sci..

[21] Gorgizadeh M., Azarpira N., Sattarahmady N. (2018). In vitro and in vivo tumor annihilation by near-infrared photothermal effect of a NiFe_2_O_4_/C nanocomposite. Colloids Surf. B Biointerfaces.

[22] Rodrigues A.R.O., Rio I.S.R., Coutinho E.M.S., Coutinho P.J.G. Development of NiFe_2_O_4_/Au nanoparticles covered with lipid bilayers for applications in combined cancer therapy. Proceedings of the Fourth International Conference on Applications of Optics and Photonics.

[23] Cabral T.C.S., Ardisson J.D., de Miranda M.C., Gomes D.A., Fernandez-Outon L.E., Sousa E.M.B., Ferreira T.H. (2019). Boron nitride nanotube@NiFe_2_O_4_: A highly efficient system for magnetohyperthermia therapy. Nanomedicine.

[24] Zhang Y., Xie M., Roscow J., Bao Y., Zhou K., Zhang D., Bowen C.R. (2017). Enhanced pyroelectric and piezoelectric properties of PZT with aligned porosity for energy harvesting applications. J. Mater. Chem. A.

[25] Roscow J.I., Lewis R.W.C., Taylor J., Bowen C.R. (2017). Modelling and fabrication of porous sandwich layer barium titanate with improved piezoelectric energy harvesting figures of merit. Acta Mater..

[26] Castro A., Morère J., Cabañas A., Ferreira L.P., Godinho M., Ferreira P., Vilarinho P.M. (2017). Designing nanocomposites using supercritical CO_2_ to insert Ni nanoparticles into the pores of nanopatterned BaTiO_3_ thin films. J. Mater. Chem. C.

[27] Castro A., Martins M.A., Ferreira L.P., Godinho M., Vilarinho P.M., Ferreira P. (2019). Multifunctional nanopatterned porous bismuth ferrite thin films. J. Mater. Chem. C.

[28] Baumgartner J., Dey A., Bomans P.H.H., Le Coadou C., Fratzl P., Sommerdijk N.A.J.M., Faivre D. (2013). Nucleation and growth of magnetite from solution. Nat. Mater..

[29] Deshpande K., Mukasyan A., Varma A. (2004). Direct synthesis of iron oxide nanopowders by the combustion approach: Reaction mechanism and properties. Chem. Mater..

[30] George C.N., Thomas J.K., Jose R., Kumar H.P., Suresh M.K., Kumar V.R., Wariar P.R.S., Koshy J. (2009). Synthesis and characterization of nanocrystalline strontium titanate through a modified combustion method and its sintering and dielectric properties. J. Alloys Compd..

[31] Leventis N., Chandrasekaran N., Sadekar A.G., Sotiriou-Leventis C., Lu H. (2009). One-pot synthesis of interpenetrating inorganic/organic networks of CuO/resorcinol-formaldehyde aerogels: Nanostructured energetic materials. J. Am. Chem. Soc..

[32] Brun N., García-González C.A., Smirnova I., Titirici M.M. (2013). Hydrothermal synthesis of highly porous carbon monoliths from carbohydrates and phloroglucinol. RSC Adv..

[33] Zhang X., Han D., Hua Z., Yang S. (2016). Porous Fe_3_O_4_ and gamma-Fe_2_O_3_ foams synthesized in air by sol-gel autocombustion. J. Alloys Compd..

[34] Xia Y., He Z., Lu Y., Tang B., Sun S., Su J., Li X. (2018). Fabrication and photocatalytic property of magnetic SrTiO_3_/NiFe_2_O_4_ heterojunction nanocomposites. RSC Adv..

[35] Kočí K., Obalová L., Matějová L., Plachá D., Lacný Z., Jirkovský J., Šolcová O. (2009). Effect of TiO2 particle size on the photocatalytic reduction of CO2. Appl. Catal. B Environ..

[36] Viennois R., Hermet P., Machon D., Koza M.M., Bourgogne D., Fraisse B., Petrović A.P., Maurin D. (2020). Stability and Lattice Dynamics of Ruddlesden–Popper Tetragonal Sr_2_TiO_4_. J. Phys. Chem. C.

[37] Ge W., Zhu C., An H., Li Z., Tang G., Hou D. (2014). Sol–gel synthesis and dielectric properties of Ruddlesden–Popper phase Sr_n+1_TinO_3n+1_ (n = 1, 2, 3, ∞). Ceram. Int..

[38] Luque R., Campelo J.M., Luna D., Marinas J.M., Romero A.A. (2007). Catalytic performance of Al-MCM-41 materials in the N-alkylation of aniline. J. Mol. Catal. A Chem..

[39] Kruk M., Jaroniec M. (2001). Gas adsorption characterization of ordered organic− inorganic nanocomposite materials. Chem. Mater..

[40] Walerczyk W., Zawadzki M., Grabowska H. (2012). Solvothermal Synthesis and Catalytic Properties of Nanocrystalline ZnFe_2−x_Al_x_O_4_ (x = 0, 1, 2) Spinels in Aniline Methylation. Catal. Letters.

[41] Li Y., Zhou X., Luo W., Cheng X., Zhu Y., El-Toni A.M., Khan A., Deng Y., Zhao D. (2019). Pore Engineering of Mesoporous Tungsten Oxides for Ultrasensitive Gas Sensing. Adv. Mater. Interfaces.

[42] Han S., Hyeon T. (1999). Simple silica-particle template synthesis of mesoporous carbons. Chem. Commun..

[43] Kushwaha S., Sreelatha G., Padmaja P. (2013). Physical and chemical modified forms of palm shell: Preparation, characterization and preliminary assessment as adsorbents. J. Porous Mater..

[44] Zeng K., Zhang D. (2010). Recent progress in alkaline water electrolysis for hydrogen production and applications. Prog. Energy Combust. Sci..

[45] Borhan A.I., Gherca D., Cojocaru Ş., Lupu N., Roman T., Zaharia M., Palamaru M.N., Iordan A.R. (2020). One-pot synthesis of hierarchical magnetic porous γ-Fe_2_O_3_@NiFe_2_O_4_ composite with solid-phase morphology changes promoted by adsorption of anionic azo-dye. Mater. Res. Bull..

[46] Yu Z., Ang C. (2002). Maxwell–Wagner polarization in ceramic composites BaTiO_3_–(Ni_0.3_Zn_0.7_)Fe_2.1_O_4_. J. Appl. Phys..

[47] Zeng F., Cao M., Zhang L., Liu M., Hao H., Yao Z., Liu H. (2017). Microstructure and dielectric properties of SrTiO_3_ ceramics by controlled growth of silica shells on SrTiO_3_ nanoparticles. Ceram. Int..

[48] Jonscher A.K. (1983). Dielectric Relaxation in Solids.

[49] Li G., Liu H., Wang Z., Hao H., Yao Z., Cao M., Yu Z. (2014). Dielectric properties and relaxation behavior of Sm substituted SrTiO_3_ ceramics. J. Mater. Sci. Mater. Electron..

[50] Lu S.G., Xu Z.K., Wang Y.P., Guo S.S., Chen H., Li T.L., Or S.W. (2008). Effect of CoFe_2_O_4_ content on the dielectric and magnetoelectric properties in Pb(ZrTi)O_3_/CoFe_2_O_4_ composite. J. Electroceramics.

[51] Bammannavar B.K., Naik L.R. (2009). Electrical properties and magnetoelectric effect in (x) Ni_0.5_Zn_0.5_ Fe_2_O ^4+(1−x)^ BPZT composites. Smart Mater. Struct..

[52] Pei Y., Li Q., Shi W., Zhang B., Chen Q., Yue X., Xiao D., Zhu J. (2010). Effect of CoFe_2_O_4_ content on the dielectric and magnetoelectric properties in CoFe_2_O_4_/Pb (Mg_1/3_Nb_2/3_)_0.35_ Ti_0.65_O_3_ composites. Ferroelectrics.

