# Supplementary material for: Sequential Synthesis Methodology Yielding Well-Defined Porous 75%SrTiO3/25%NiFe2O4 Nanocomposite
Source: Nanomaterials (Basel). 2021 Dec 31;12(1):138. doi: 10.3390/nano12010138 (PMC8747004; doi:10.3390/nano12010138)
Supplement: Supplementary file 1 [file nanomaterials-12-00138-s001.zip › nanomaterials-1507084-supplementary.pdf]

Supporting Information

Sequential Synthesis Methodology Yielding Well-Defined Porous $_{75\%}SrTiO_3/_{25\%}NiFe_2O_4$ Nanocomposite

Ilyes Baba-Ahmed [1], Daniel Ghercă [2†], Alexandra-Raluca Iordan [3], Mircea Nicolae Palamaru [3], Carmen Mita [3], Mohamed Amine Benamar [4], Gabriel Ababei [2], Nicoleta Lupu [2], Rachid Baghdad [5], Abdelkader Abderrahmane [6], Tiberiu Roman [7], Georgiana Bulai [7], Liviu Leontie [8] and Adrian Iulian Borhan [2,3,*,†]

1 Laboratory of Fundamental and Applied Physics (FUNDAPL), Physics Department, Sciences Faculty, Saad Dahleb Blida 1 University, BP 270, Blida 09000, Algeria; baba.ing.ebm@gmail.com
2 National Institute of Research and Development for Technical Physics, 47 Mangeron Boulevard, 700050 Iasi, Romania; dgherca@phys-iasi.ro (D.G.); gababei@phys-iasi.ro (G.A.); nicole@phys-iasi.ro (N.L.)
3 Faculty of Chemistry, Alexandru Ioan Cuza University of Iasi, 11 Carol I Boulevard, 700506 Iasi, Romania; alexandra.iordan@uaic.ro (A.R.I.); palamaru@uaic.ro (M.N.P.); cmita@uaic.ro (C.M.)
4 Laboratory of Fundamental and Applied Physics (FUNDAPL), University Center of Tamenghasset, Amine Elokkal Elhadj Moussa Eg-Akhamouk, BP10034, Sersouf, Tamanghasset, 11000, Algeria; benamardz64dz@yahoo.fr
5 Synthesis and Catalysis Laboratory, Matter Sciences,Faculty, Ibn Khaldoun University of Tiaret, Tiaret 14000, Algeria; baghdadrachid@gmail.com
6 Department of Electrical Engineering, Chosun University, 375, Seosuk-dong, Dong-gu, Gwangju 501759, Korea; abderrahmane.abdelkader@gmail.com
7 Integrated Center of Environmental Science Studies in the North-Eastern Development Region (CERNESIM), Department of Exact and Natural Sciences, Institute of Interdisciplinary Research, Alexandru Ioan Cuza University of Iasi, 700506 Iasi, Romania; tiberiu.roman@chem.uaic.ro (T.R.); georgiana.bulai@uaic.ro (G.B.)
8 Faculty of Physics, Alexandru Ioan Cuza University of Iasi, 11 Carol I Boulevard, 700506 Iasi, Romania; lleontie@uaic.ro
* Correspondence: adrian.borhan@uaic.ro
† These authors contributed equally to this work.

Citation: Baba-Ahmed, I.; Ghercă, G.; Iordan, A.R.; Palamaru, M.N.; Mita, C.; Benamar, M.A.; Ababei, G.; Lupu, N.; Baghdad, R; Abderrahmane, A.; et al. Sequential Synthesis Methodology Yielding Well-Defined Porous $_{75\%}SrTiO_3/_{25\%}NiFe_2O_4$ Nanocomposite. *Nanomaterials* **2022**, *11*, 138. https://doi.org/10.3390/nano12010138

Academic Editor(s): Yun-Mo Sung

Received: date
Accepted: date
Published: date

Publisher's Note: MDPI stays neutral with regard to jurisdictional claims in published maps and institutional affiliations.

Copyright: © 2021 by the authors. Submitted for possible open access publication under the terms and conditions of the Creative Commons Attribution (CC BY) license (https://creativecommons.org/licenses/by/4.0/).

The crystal structure and crystallinity of nanopowders were analyzed by means of X-ray diffraction analysis performed at room temperature.

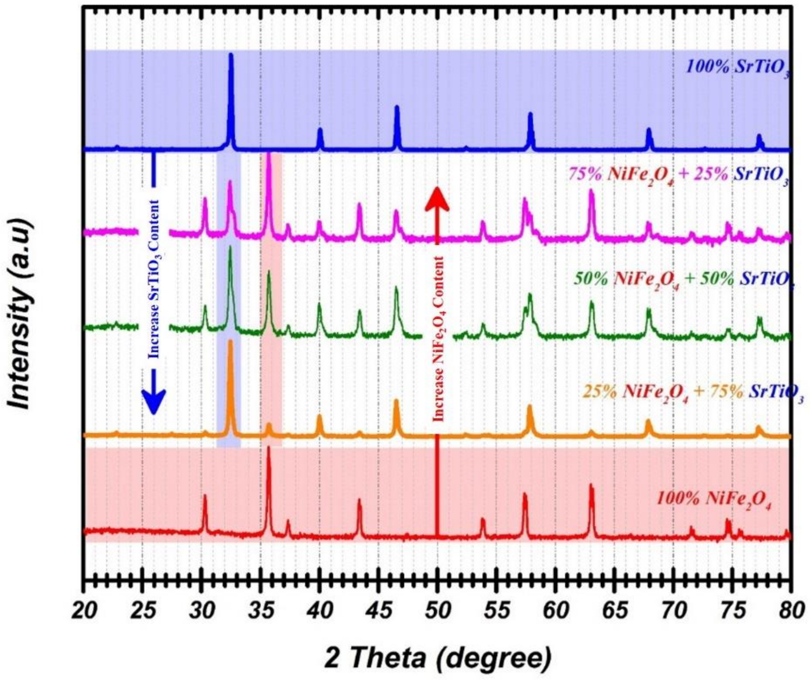

Figure S1. The superimposed XRD patterns of perovskite phases ($SrTiO_3$) synthesized with different ratios of spinel phase ($NiFe_2O_4$).

The XRD patterns Figure S2-S6 shows X-ray Rietveld refinement carried out using the FullProf 2000 program by profile fitting, with a peak shape modelled by a Pseudo-Voigt function using the Thomson Cox Hastings model.

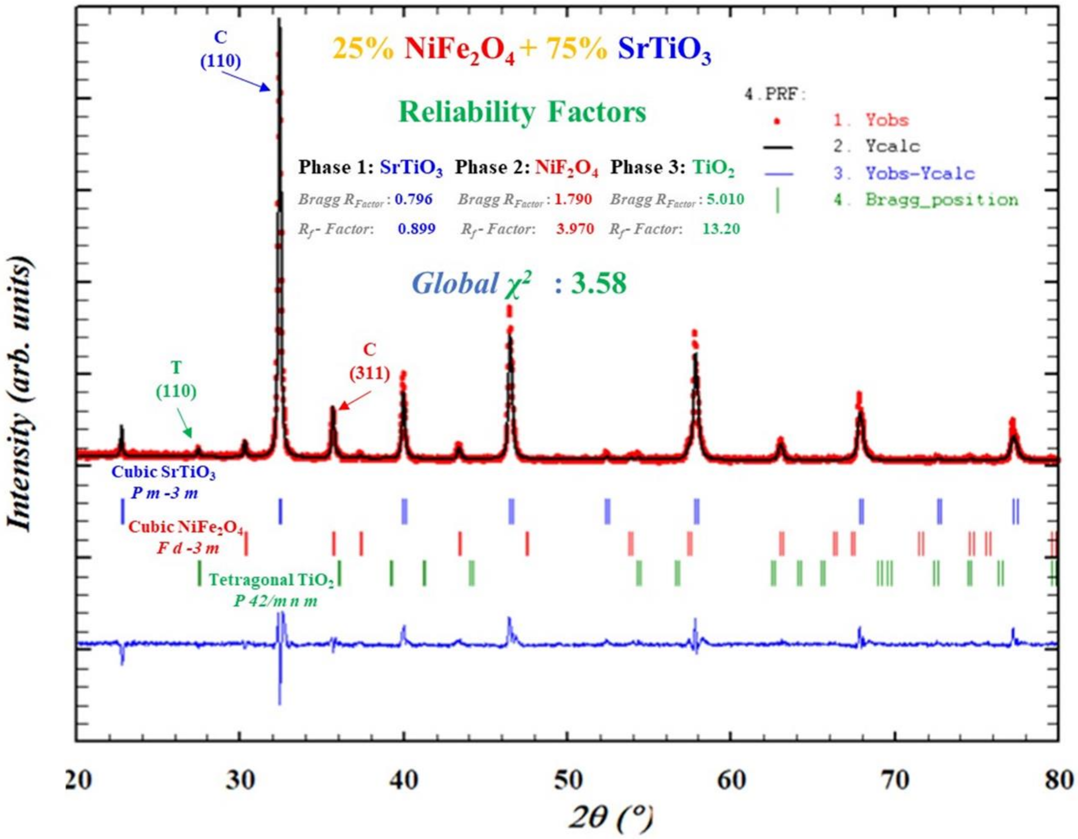

Figure S2. The Rietveld refinement of the $_{75}$STO/$_{25}$NFO. Black solid line is the best fits to the experimental data, the blue line represents the differences between the experimental and calculated data while the vertical markers represent the Bragg peaks positions in blue for Cubic perovskite phase, in red for Cubic spinel phase and in green for Tetragonal TiO_2 phase.

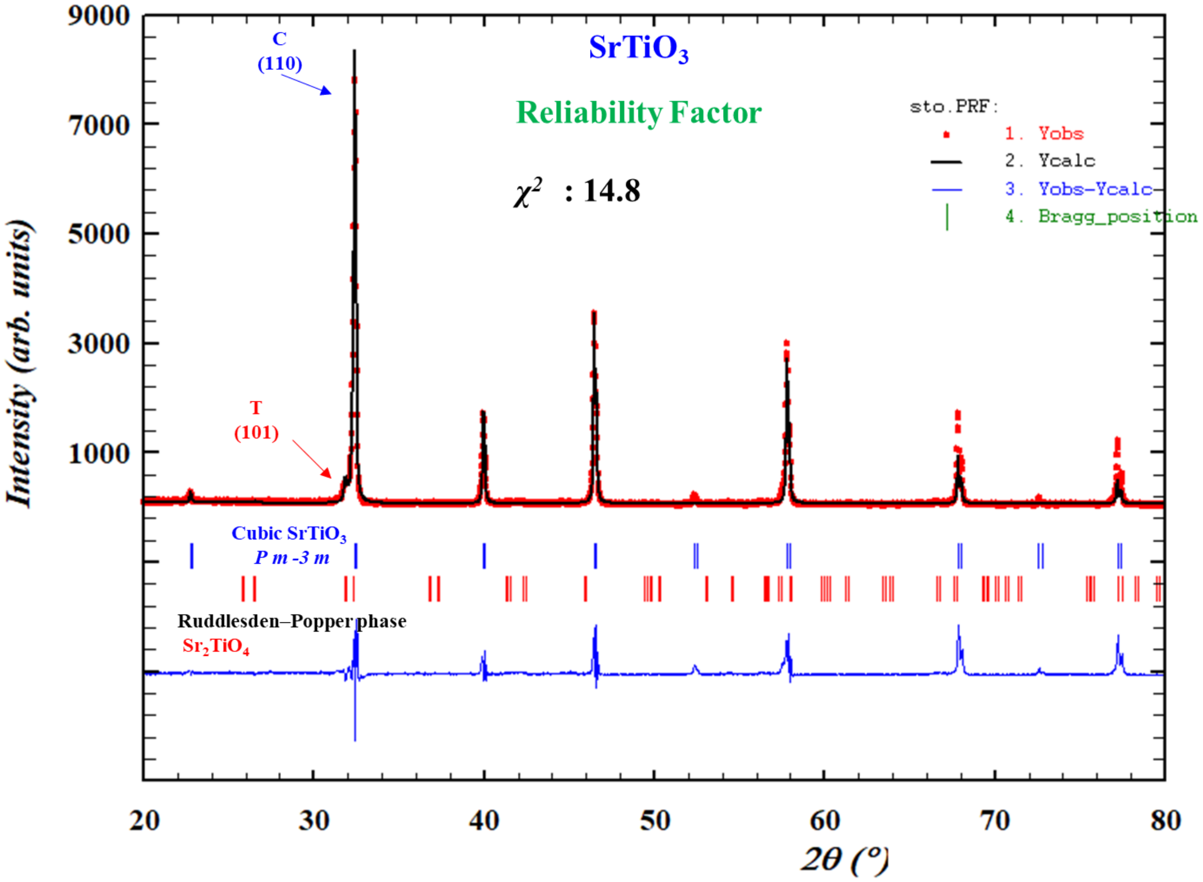

Figure S3. Le Bail refinement from laboratory XRD patterns at RT The Rietveld refinement of $SrTiO_3$ nanoparticles. Black solid line is the best fits to the experimental data, the blue line represents the differences between the experimental and calculated data while the vertical markers represent the Bragg peaks positions in blue for Cubic perovskite phase and in red for Ruddlesden–Popper phase.

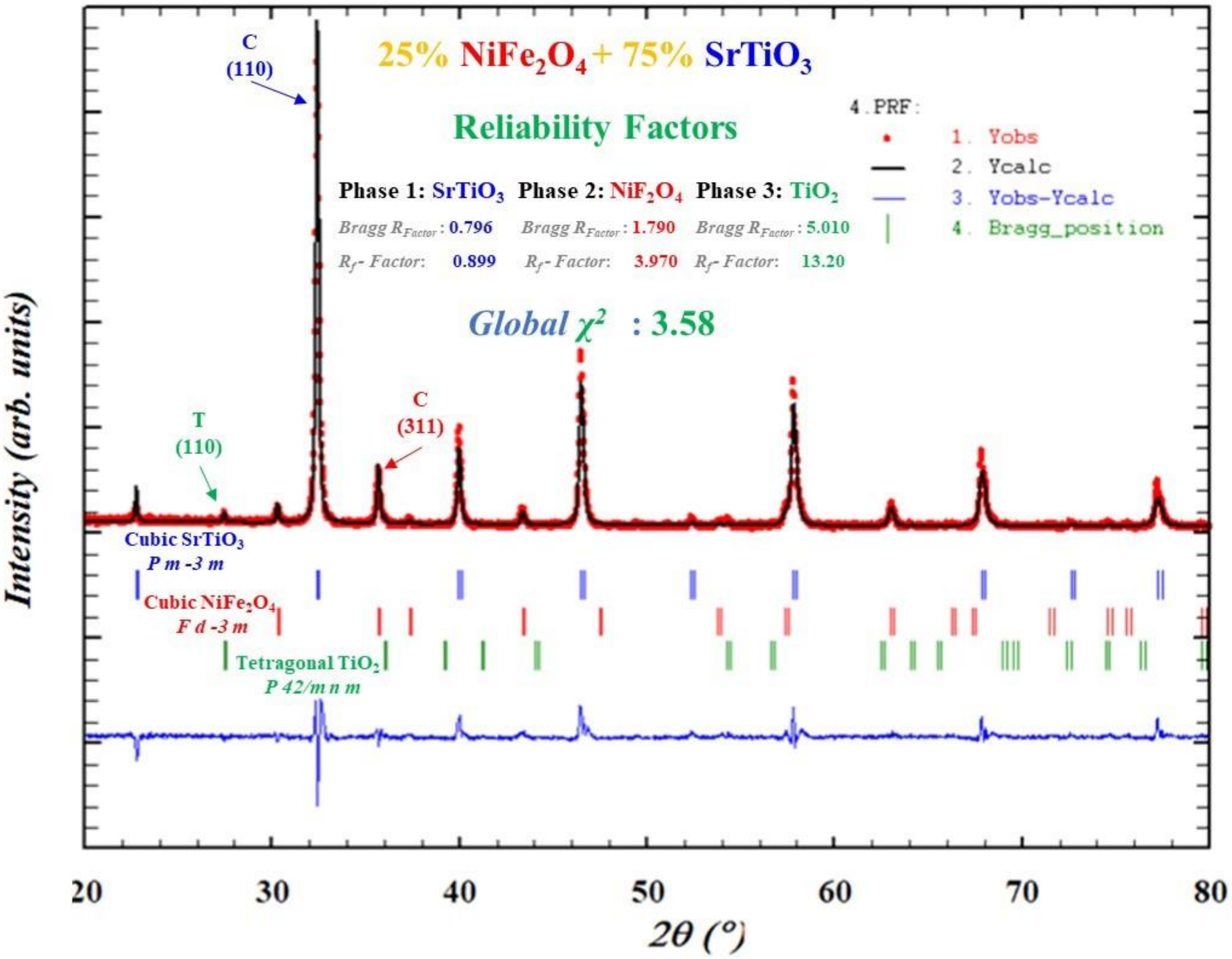

Figure S4. The Rietveld refinement of $SrTiO_3$ with 25% of $NiFe_2O_4$. Black solid line is the best fits to the experimental data, the blue line represents the differences between the experimental and calculated data while the vertical markers represent the Bragg peaks positions in blue for Cubic perovskite phase and in red for Cubic spinel phase and in green for Tetragonal TiO_2 phase.

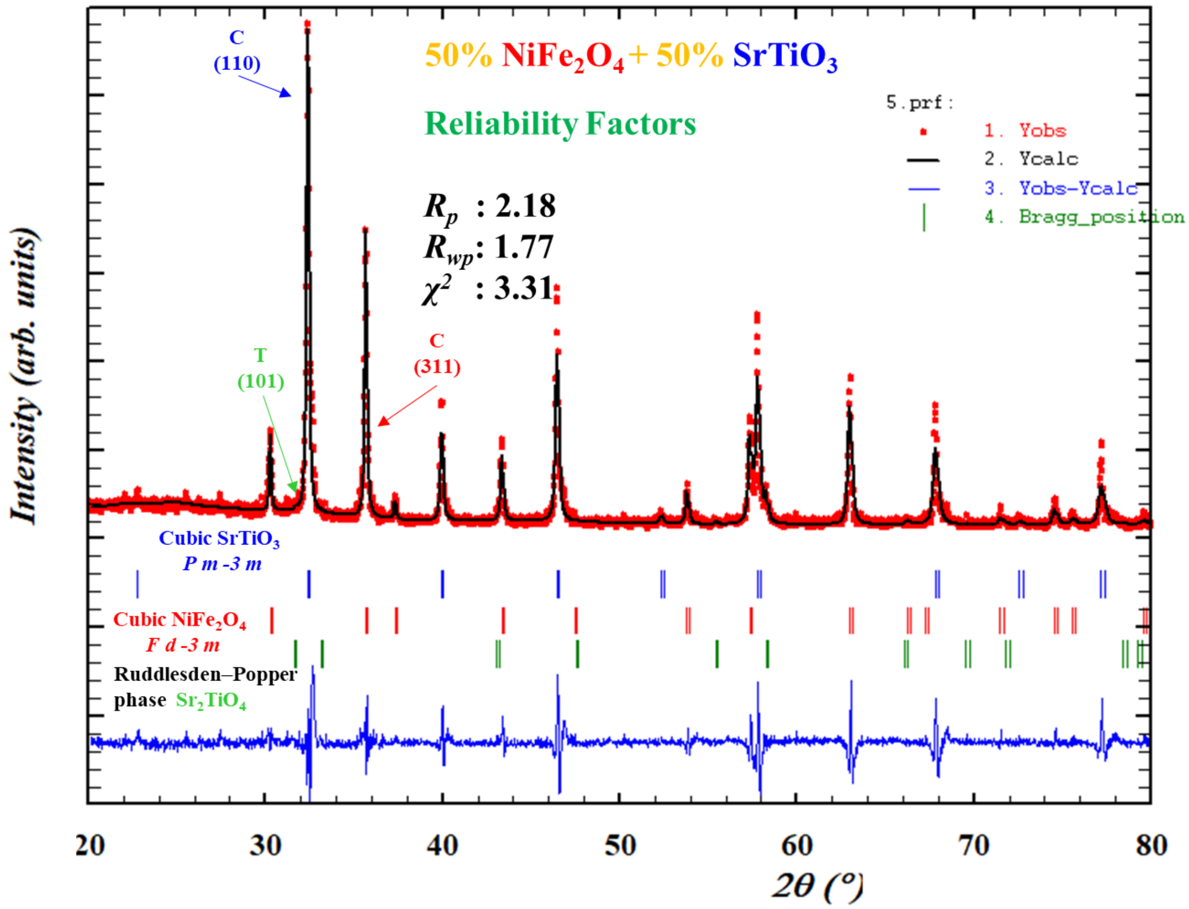

Figure S5. The Rietveld refinement of $SrTiO_3$ with 50% of $NiFe_2O_4$. Black solid line is the best fits to the experimental data, the blue line represents the differences between the experimental and calculated data while the vertical markers represent the Bragg peaks positions in blue for Cubic perovskite phase, in green for Tetragonal perovskite phase and in red Cubic spinel phase.

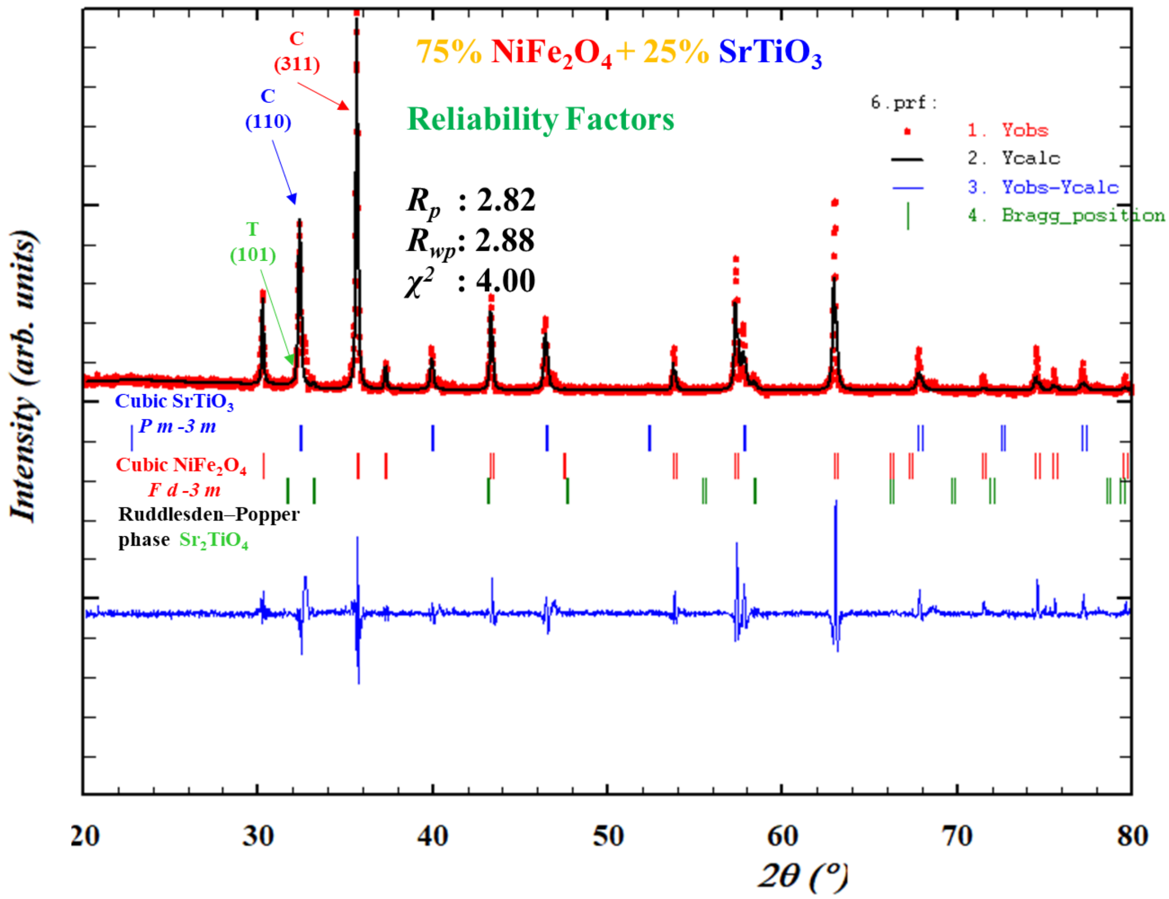

Figure S6. The Rietveld refinement of $SrTiO_3$ with 75% of $NiFe_2O_4$. Black solid line is the best fits to the experimental data, the blue line represents the differences between the experimental and calculated data while the vertical markers represent the Bragg peaks positions in blue for Cubic perovskite phase, in green for Tetragonal perovskite phase and in red Cubic spinel phase.

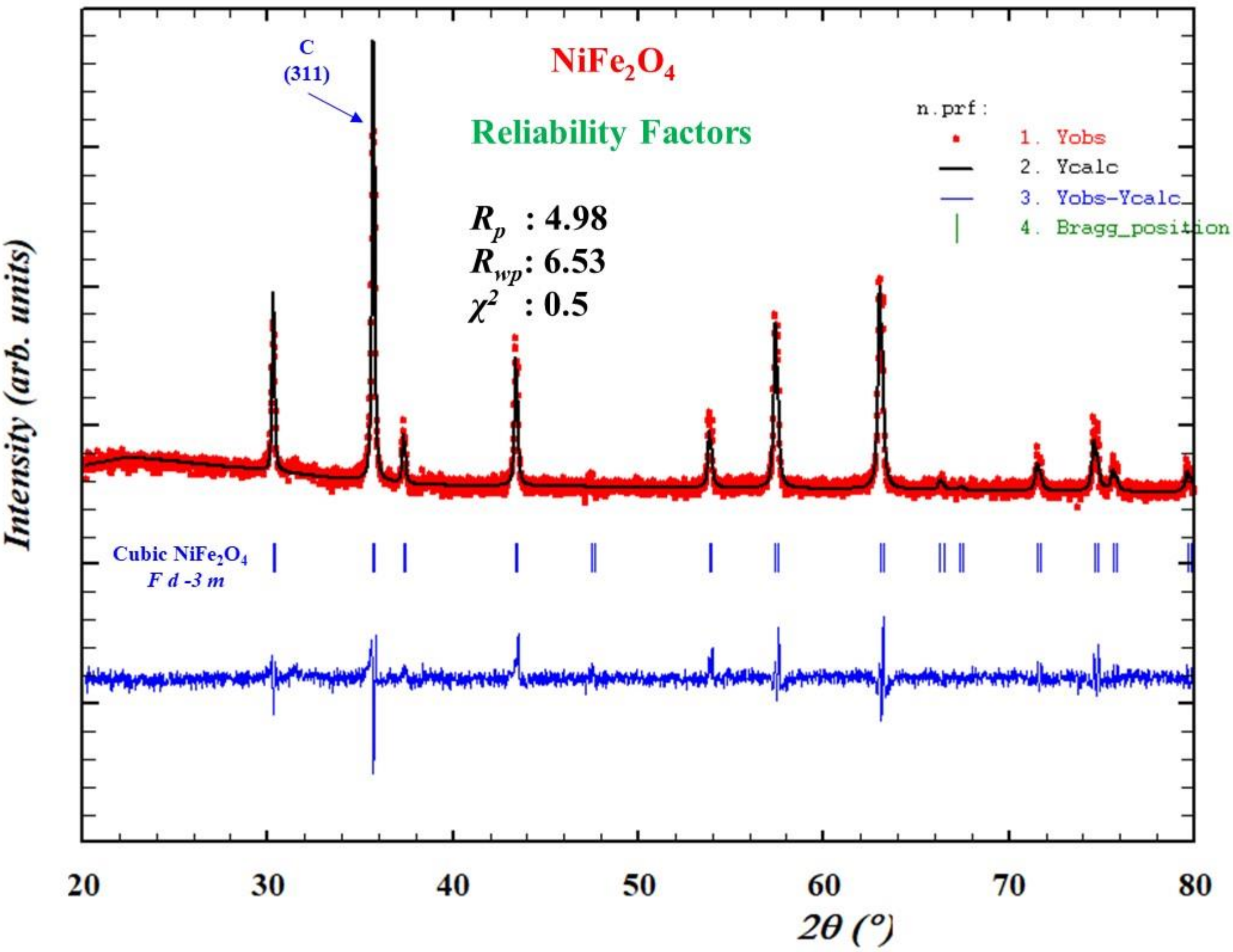

Figure S7. The Rietveld refinement of $NiFe_2O_4$ nanoparticles. Black solid line is the best fits to the experimental data, the blue line represents the differences between the experimental and calculated data while the vertical markers represent the Bragg peaks positions in blue for Cubic spinel phase.

As examined in Figure 4(a), the typical TEM images not only certify the distinctive crystalline phases obtained $NiFe_2O_4$ and $SrTiO_3$ respectively but also reveal the interconnection between them; it shows clearly that $SrTiO_3$ and $NiFe_2O_4$ nanoparticles are closely connected with each other. That is, the nanocomposite can be considered as the famous heterojunction connection by sequential synthesis methodology addressed in this study which enables $_{75}$STO/$_{25}$NFO successfully fabrication as porous-foam in nature by joint solid-state reaction and sol-gel auto-combustion technique. Figure 4(b) – (d) reveals that consolidation of particles occurs by exact stacking of $NiFe_2O_4$ particles along with crystal facets of $SrTiO_3$. The fact of crystal facets of perovskite phase might be the key role in the maintaining and formation of such hierarchical nano heterostructures proved by powerful evidence Figure 4 (b) – (d).

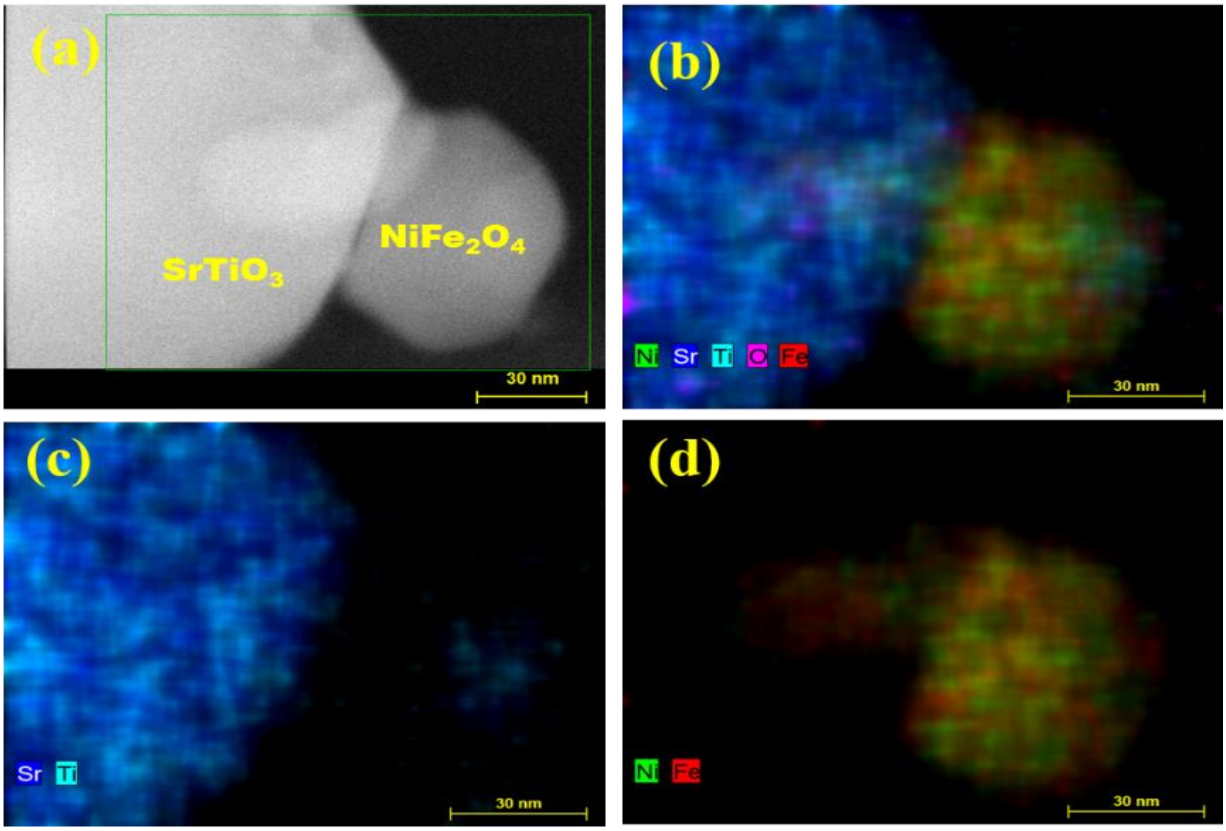

Figure S8. TEM dark field image of $NiFe_2O_4$ particle attachment on $SrTiO_3$ polyhedral nanoparticles (a) and the corresponding elemental mapping for all elements Fe, Ni, Sr, Ti, O (b) Sr, Ti (c) and Fe, Ni (d).
